# Jak2V617F myeloproliferative neoplasm stem cells and interferon-alpha

**DOI:** 10.18632/oncotarget.986

**Published:** 2013-04-21

**Authors:** Steven W. Lane, Ann Mullally

**Affiliations:** Queensland Institute of Medical Research, University of Queensland, Brisbane, Australia; Division of Hematology, Brigham and Women's Hospital, Harvard Medical School, Boston, MA.

The myeloproliferative neoplasms (MPNs) are clonal disorders of hematopoiesis that arise as a result of aberrant activation of tyrosine kinases and result in the proliferation and accumulation of mature myeloid cells in the blood, bone marrow and spleen. The prototypical MPN, chronic myeloid leukemia (CML) is caused by constitutive activation of ABL kinase occurring as a result of the *BCR-ABL* fusion. Importantly, targeted therapy to inhibit ABL kinase signaling, using imatinib, induces durable clinical responses in the majority of CML patients. The molecular pathogenesis of the BCR-ABL negative MPNs, polycythemia vera (too many red cells), essential thrombocythemia (too many platelets) and myelofibrosis, was elucidated in 2005 with the discovery of JAK2V617F, an activating mutation in the non-receptor tyrosine kinase, JAK2 [1]. The JAK2V617F mutation is present in more than 95% patients with polycythemia vera, and approximately 50% patients with essential thrombocythemia and myelofibrosis. In advanced disease, MPN may transform to acute leukaemia and this is associated with a dismal prognosis.

In MPN, JAK2V617F is found in long-term hematopoietic stem cells (LT-HSCs), a population of rare quiescent cells present in the bone marrow and capable of long-term self-renewal. These somatically mutated LT-HSCs, referred to as MPN-propagating cells or MPN stem cells, maintain the MPN throughout the life of the patient and can serially transplant disease in animal models of Jak2V617F-mediated MPN [2, 3]. To definitively cure MPN will require complete eradication of the MPN disease-maintaining stem cell population. In contrast to the efficacy of imatinib in achieving molecular remissions in CML, JAK2 kinase inhibitors have shown only modest benefit in the treatment of myelofibrosis, namely in reducing splenomegaly and improving constitutional symptoms. Furthermore, the lack of an effect of the JAK2 inhibitors on reducing *JAK2V617F* allele burden in myelofibrosis patients [4] and the demonstration that MPN-propagating cells are insensitive to JAK2 kinase inhibition in vivo [2] suggests that MPN stem cells do not require JAK2 signaling for survival and that JAK2 inhibitor monotherapy will not cure MPN patients.

The clinical efficacy of long-acting pegylated IFNα in MPN has renewed interest in understanding the mechanism of action of IFNα treatment in *JAK2V617F*-mediated MPN. Prolonged IFNα treatment results in normalization of blood counts in most patients with polycythemia vera, reduces *JAK2V617F* allele burden and in 15-40% of patients, the *JAK2V617F* mutant clone is rendered undetectable to sensitive molecular assays[5, 6]. These clinical results suggest that IFNα eradicates JAK2V617F-mutant LT-HSC, however the mechanism by which molecular remissions are achieved in MPN patients in response to treatment with IFNα have not been well defined.

**Figure 1:**
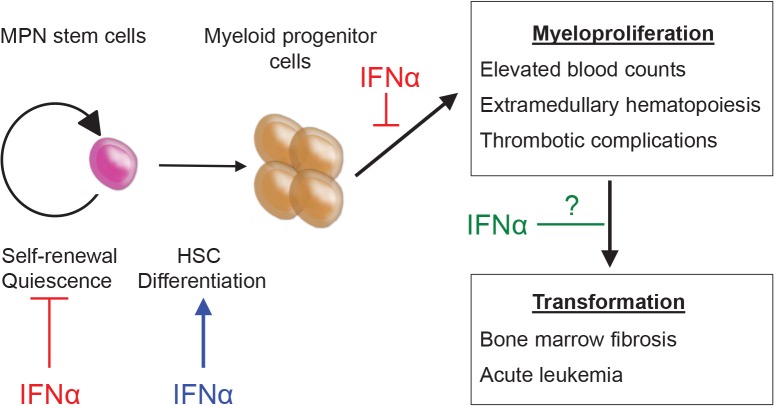
MPN stem cells are responsible for disease initiation and propagation in vivo IFNα therapy has direct effects on MPN stem cells leading to depletion of LT-HSCs, exit from quiescence and enforced terminal differentiation. Additionally, IFNα may have direct effects on downstream effector cells leading to reduction in blood counts and extramedullary hematopoiesis. The effects of IFNα on preventing fibrotic and/or leukemic transformation have not yet been defined

To determine the efficacy of IFNα therapy on Jak2V617F-positive MPN stem cells *in vivo*, we used a murine model of Jak2V617F-mediated MPN [7]. We found that prolonged treatment of Jak2V617F mice with IFNα reversed the myeloproliferation phenotype (normalizing hemoglobin and white cell count and reducing extramedullary hematopoiesis). Importantly, we observed selective depletion of Jak2V617F over WT LT-HSC and this correlated with an inability of IFNα treated mice to propagate MPN into transplanted recipients. IFNα can directly activate the cell cycle in quiescent wild-type (WT) LT-HSCs and induce HSC proliferation *in vivo [8]*. We found that IFNα treatment induced enhanced cell-cycle activation of Jak2V617F-mutant LT-HSCs, as compared to WT LT-HSCs, explaining this preferential depletion over time. Using Ifnar1 knock-out mice (lacking the type 1 interferon receptor), we showed that the effects of IFNα on cell cycle are specifically mediated through type 1 interferon signaling and are intrinsic to Jak2V617F-mutant LT-HSCs. Using gene expression analysis we found an enrichment of cell cycle genes in Jak2V617F-mutant LT-HSC, both at baseline and after IFNα treatment, providing a potential explanation for their preferential sensitivity to IFNα treatment.

This work suggests that IFNα achieves molecular remissions in JAK2V617F-positive MPN patients through the activation and consequent depletion of quiescent MPN disease-propagating cells. What are the clinical implications of this work? Firstly, these data support the rational design of clinical trials to test combinatorial therapeutic approaches using IFNα (to target quiescent MPN-stem cells) together with JAK2 kinase inhibitors or cytotoxic chemotherapy (to target actively cycling progenitor populations). Plausibly, the depletion of MPN stem cells in polycythemia vera could alter the natural history of the disease, through eliminating the malignant clone and thus preventing fibrotic and/or leukemic transformation, both devastating complications of MPN. Currently, IFNα is far from an ideal drug, and few patients are able to tolerate prolonged treatment due to its side effects. We have identified a number of novel targets that are differentially regulated in Jak2V617F and WT LT-HSCs following IFNα treatment. In ongoing studies, we are working to validate these targets with the goal of developing more specific, less toxic IFNα derivatives capable of selectively eliminating MPN stem cells, while sparing normal LT-HSCs and other organ systems.
